# Albino mice with the point mutation at the tyrosinase locus show high cholesterol diet-induced NASH susceptibility

**DOI:** 10.1038/s41598-021-00501-5

**Published:** 2021-11-08

**Authors:** Kaushalya Kulathunga, Arata Wakimoto, Yukiko Hiraishi, Manoj Kumar Yadav, Kyle Gentleman, Eiji Warabi, Tomoki Sakasai, Yoshihiro Miwa, Seiya Mizuno, Satoru Takahashi, Michito Hamada

**Affiliations:** 1 Department of Anatomy and Embryology, Faculty of Medicine, University of Tsukuba, 1-1-1, Tennodai, Tsukuba, Ibaraki 305-8575 Japan; 2 Laboratory Animal Resource Center, Faculty of Medicine, University of Tsukuba, 1-1-1, Tennodai, Tsukuba, Ibaraki 305-8575 Japan; 3 Integrated Master of Science Natural Sciences, University of Southampton, Highfield, Southampton, Hampshire SO17 1BJ UK; 4 International Institute for Integrative Sleep Medicine (WPI-IIIS), University of Tsukuba, 1-1-1, Tennodai, Tsukuba, Ibaraki 305-8575 Japan; 5 Department of Physiology, Faculty of Medicine, Sabaragamuwa University of Sri Lanka, Hidellana, P.O. Box 01, Ratnapura, Sri Lanka; 6 Gene Engineering Division, BioResource Research Center, RIKEN, 3-1-1, Koyadai, Tsukuba, Ibaraki 305-0074 Japan

**Keywords:** Mechanisms of disease, Fat metabolism

## Abstract

Non-alcoholic fatty liver disease (NAFLD) constitutes a metabolic disorder with high worldwide prevalence and increasing incidence. The inflammatory progressive state, non-alcoholic steatohepatitis (NASH), leads to liver fibrosis and carcinogenesis. Here, we evaluated whether tyrosinase mutation underlies NASH pathophysiology. Tyrosinase point-mutated B6 (Cg)-*Tyr*^*c-2J*^/J mice (B6 albino) and C57BL/6J black mice (B6 black) were fed with high cholesterol diet (HCD) for 10 weeks. Normal diet-fed mice served as controls. HCD-fed B6 albino exhibited high NASH susceptibility compared to B6 black, a phenotype not previously reported. Liver injury occurred in approximately 50% of B6 albino from one post HCD feeding, with elevated serum alanine aminotransferase and aspartate aminotransferase levels. NASH was induced following 2 weeks in severe-phenotypic B6 albino (sB6), but B6 black exhibited no symptoms, even after 10 weeks. HCD-fed sB6 albino showed significantly higher mortality rate. Histological analysis of the liver revealed significant inflammatory cell and lipid infiltration and severe fibrosis. Serum lipoprotein analysis revealed significantly higher chylomicron and very low-density lipoprotein levels in sB6 albino. Moreover, significantly higher small intestinal lipid absorption and lower fecal lipid excretion occurred together with elevated intestinal NPC1L1 expression. As the tyrosinase point mutation represents the only genetic difference between B6 albino and B6 black, our work will facilitate the identification of susceptible genetic factors for NASH development and expand the understanding of NASH pathophysiology.

## Introduction

Non-alcoholic fatty liver disease (NAFLD) is a pathological condition characterized by increased lipid accumulation in liver hepatocytes without concomitant alcohol abuse or other liver diseases. NAFLD progression into the inflammatory state, termed non-alcoholic steatohepatitis (NASH), leads to more serious presentation, including hepatic inflammation, hepatocyte damage, and fibrosis^1^. NASH also increases the risk of developing cirrhosis or hepatocellular carcinoma^2^. The estimated worldwide prevalence of NAFLD is approximately 25%^3^, with 10–30% of cases progressing into NASH^4^.

NAFLD is frequently associated with obesity, diabetes, dyslipidemia, and hypertension and is considered as the liver manifestation of metabolic syndrome^5^. Current evidence suggests that NAFLD exhibits multifactorial pathophysiology^6^ with metabolic syndrome, genetic factors, oxidative stress, inflammatory cytokines, endotoxins, and insulin resistance contributing to disease development. However, the detailed molecular mechanisms involved in disease progression to NASH remain unclear, in part because of the lack of appropriate animal models.

Dietary cholesterol contributes to the development of steatohepatitis in both animal models and humans, facilitating NASH development by deregulating inflammation and metabolism-associated gene expression^7^. Extensive deregulation of hepatic cholesterol homeostasis in NAFLD leads to increased hepatic free cholesterol levels^8^; these are implicated in hepatic lipotoxicity^9^, thereby activating inflammatory recruitment to fatty livers to induce NASH. This can initiate with cholesterol crystal accumulation and hepatic crown-like structure formation^10^, plus cholesterol-induced Kupffer cell and NLRP3 inflammasome activation^11,12^. Thus, cholesterol rather than triglyceride and free fatty acid accumulation may constitute an important risk factor of NASH development.

The cholesterol level in the body comprises liver-biosynthesized “endogenous cholesterol” and dietary “exogenous cholesterol”^13^, with dietary cholesterol playing a more substantial role in NASH development^14^. These findings highlight the small intestines (SI) as another important regulator of whole-body cholesterol homeostasis. Genetic deletion of Niemann-Pick C1 like 1 protein (NPC1L1), which regulates dietary cholesterol absorption by the SI^15^ reduces cholesterol absorption by > 70% in mice^16^. Alternatively, ATP-binding cassette ABCG5/ABCG8 heterodimer is crucial for intestinal cholesterol excretion^17^. Nevertheless, a comprehensive profile of intestinal cholesterol regulation remains to be elucidated.

Racial/ethnic disparities in NAFLD prevalence and severity are widely documented. Clarifying the factors underlying these differences may increase understanding of disease pathophysiology. NAFLD prevalence is highest in Hispanics (22.9%), intermediate in whites (14.4%), and lowest in blacks (13%)^18^. NASH risk is also greater among Hispanic patients with NAFLD^19^. Notably, in preliminary in silico analyses we observed that frequencies of single nucleotide polymorphisms (SNPs) in the tyrosinase (*Tyr*) gene also differ among ethnic groups with two main SNPs, G to A at nt 140^20^ and G to C at nt 316^21^, exhibiting relatively high allele frequencies in Hispanic populations^22^. Therefore, here we aimed to investigate whether *Tyr* gene variants enhance NAFLD/NASH susceptibility and severity.

Specifically, we evaluated the effects of a high cholesterol diet (HCD) in B6 albino mice, which carry a *Tyr* SNP, leading to melanin synthesis deficiency and white coat color^23^, along with standard C57BL/6 (B6 black) mice. It has been reported that HCD induces steatohepatitis in mice^14^. Matsuzawa et al. found that an atherogenic diet containing 1.25% cholesterol increased the expression levels of genes related to fatty acid synthesis, oxidative stress, inflammation, and fibrogenesis in the liver^14^. Therefore, we used this HCD to evaluate the characteristics and timing of NASH development including fatty liver and fibrotic phenotype, measuring liver inflammation and lipid deposition along with cholesterol crystal formation and fibrosis. Comparison with human NASH-like disease progression was assessed regarding phenotypical lipid deposition, hepatocyte ballooning, hepatic crown-like structure, inflammation, and rapid fibrosis. We found that SNPs in *Tyr* may constitute risk factors in NAFLD/NASH susceptibility and severity, representing novel genetic contribution toward NAFLD/NASH pathophysiology and suggest that this rapid model of diet-induced NASH with fibrosis may surpass the limitation of existing NASH models.

## Results

### B6 albino mice develop NASH upon HCD feeding

**Figure 1:**
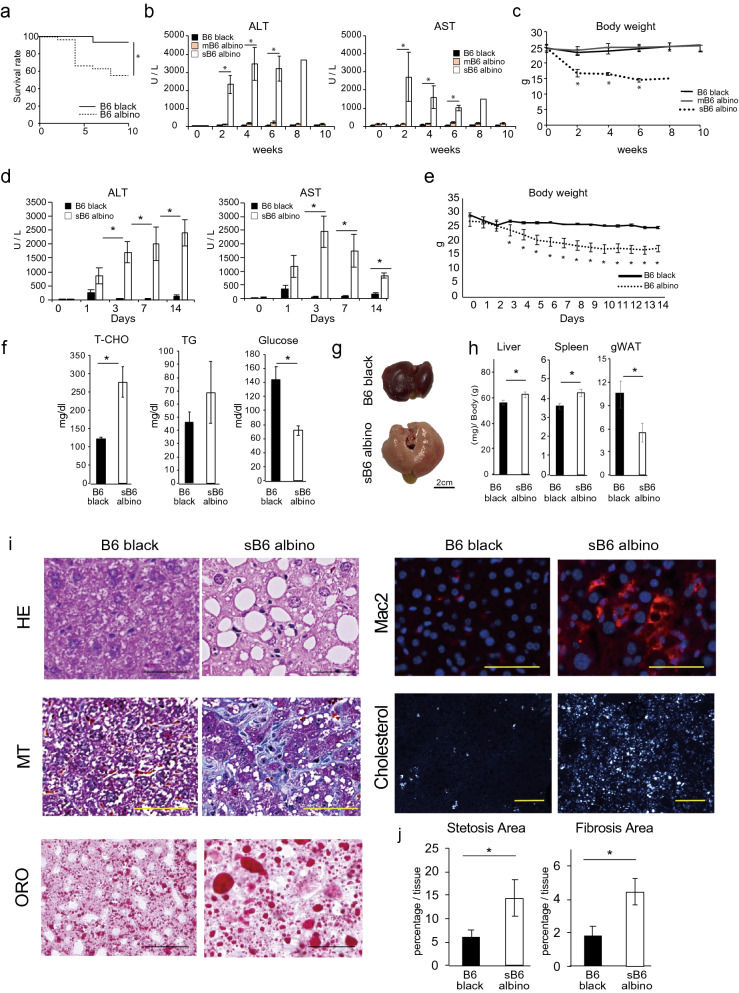
Severe B6 (sB6) albino mice develop NASH with 2 weeks of HCD feeding. (**a**) Survival curve of HCD fed B6 albino (dotted line, n = 27) and B6 black (thick line, n = 17) under HCD feeding. *p < 0.05 B6 albino compared with B6 black (log-rank test). (**b**) Serum ALT, AST levels of HCD-fed B6 black, mild B6 (mB6) albino and sB6 albino for 10 weeks. (**c**) Body weight of HCD-fed B6 albino and B6 black for 10 weeks. All the data are presented as the mean ± s.e.m. *p < 0.05 sB6 albino compared with B6 black (Welch’s t-test). (**d**) Serum ALT, AST levels of HCD-fed B6 black, mild B6 (mB6) albino and severe B6 (sB6) albino mice for 14 days. (**e**) Body weight of 2 weeks HCD-fed sB6 albino and B6 black. B6 black (n = 3), sB6 albino (n = 6) mice. (**f**) Serum total cholesterol (T-CHO), triglyceride (TG) and glucose levels of 2 weeks HCD-fed B6 black (n = 3) and sB6 albino (n = 6) mice. (**g**) Representative macroscopic appearance of the liver. (**h**) Relative weight of the liver (B6 black n = 14, sB6 albino n = 25), spleen (n = 14, n = 25), and gWAT (n = 9, n = 19) after 2 weeks of HCD feeding. (**i**) Representative microscopic view of liver sections observed using HE, MT, ORO, anti- mouse Mac2 staining, and under polarized light (Scale bar = 50 μm). (**j**) Quantified area of liver section steatosis and fibrosis (B6 black n = 3, sB6 albino n = 6). The data are from one representative experiment of at least two independent experiments and are presented as the means ± s.e.m. *p < 0.05 compared with B6 black (Welch’s t-test).

To evaluate the high cholesterol effect on a mouse, B6 albino and B6 black male mice (10–12 weeks-old) were fed with 1.25% HCD for 10 weeks. HCD feeding yielded significantly higher mortality in B6 albino than B6 black (Fig. 1a) with 12/27 vs. 1/17 respective mouse deaths starting from week 2 of HCD feeding. Overall, approximately 50% of B6 albino died throughout the experiments performed. Serum ALT and AST levels were checked biweekly to identify the underlying cause for the severe cachexia and death. Though these levels did not differ between the groups prior to HCD feeding, we observed that the B6 albino could be divided into two groups, one in which their values are increased 20-fold compared to B6 black (Severe B6 albino: sB6 albino) and the other in which their levels are almost the same (Mild B6 albino: mB6 albino) (Fig. 1b). Severe B6 albino presented 40% body weight reduction upon HCD feeding compared to mB6 albino and B6 black (Fig. 1c). The results of the initial 2-week stage following HCD feeding revealed that serum ALT and AST measurements were elevated at day 1 and significantly elevated at days 3, 7, and 14 in sB6 albino (Fig. 1d) along with significantly decreased body weight (Fig. 1e).

Notably, serum cholesterol levels before HCD induction significantly differed between sB6 albino and mB6 albino upon HCD feeding, with the former displaying significantly lower serum T-CHO levels at day 0 (Supplementary Fig. 1a,b). Pre-HCD serum cholesterol and day 14 post-HCD serum ALT showed a significant negative correlation (Supplementary Fig. 1b); this pattern was not observed in B6 black. This phenomenon thus allowed prediction and pre-selection of sB6 albino that would develop NASH phenotype upon HCD feeding. Subsequent experiments focused on the sB6 albino.

Altered serum parameters were observed at both 2 and 10 weeks of HCD feeding, with two-fold significant increase in serum T-CHO, increased serum triglyceride (TG), and significant 50% reduction in serum glucose levels, compared with B6 black (Fig. 1f, Supplementary Fig. 2a). Macroscopic analysis after 2 and 10 weeks revealed pale color, enlarged livers in sB6 albino whereas B6 black mouse livers were normal in size and appearance (Fig. 1g, Supplementary Fig. 2b). Severe B6 albino presented significant hepatomegaly and splenomegaly albeit significantly reduced white adipose tissue (WAT) content, with gonadal WAT (gWAT) being totally absent in some animals (Fig. 1h, Supplementary Fig. 2c). Extensive histological analysis revealed severe changes in liver tissue morphology with accumulated lipid droplets, fibrosis, macrophage infiltration, and cholesterol crystal accumulation in sB6 albino livers whereas B6 black livers only showed mild microvascular lipid deposition (Fig. 1i,j, Supplementary Fig. 3a,b). Presence of elevated intracellular free cholesterol was confirmed using Filipin staining (Supplementary Fig. 4a,b). These histological findings closely resemble the clinical features of NASH and confirm that HCD feeding induces rapid development of NASH-like phenotype in sB6 albino in just 2 weeks.

To further confirm induced inflammation in sB6 albino liver tissue, expression of the pro-inflammatory mediator *Il1b*^24^ was measured in liver tissue using RT-qPCR. sB6 albino liver tissue exhibited threefold elevated Il1b expression, confirming severe inflammation (Supplementary Fig. 4c, left panel). Expression levels of fibrosis-related genes *Tgfb* and *Col1al* were three-fold higher in sB6 albino than B6 black mouse liver tissue, confirming histological observations of severe fibrosis (Supplementary Fig. 4c, middle and right panel).

### HCD-fed sB6 albino exhibit rapid NASH phenotype onset

**Figure 2:**
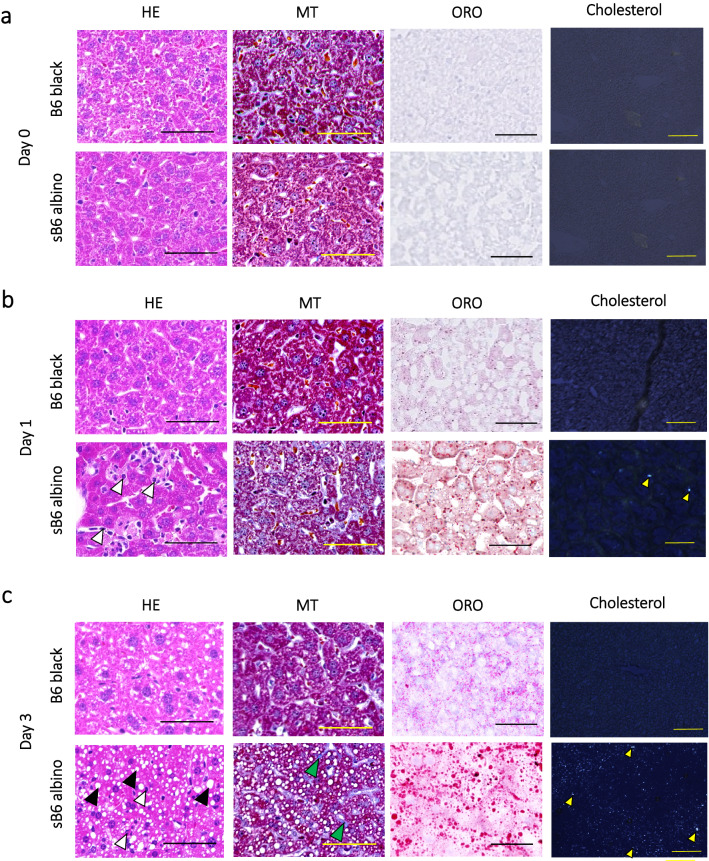
Rapid onset of NASH phenotype in sB6 albino mice upon HCD induction. (**a**) Representative microscopic images of liver tissues before HCD (**a**), 1-day (**b**), and 3-days (**c**) after HCD feeding. Liver sections were stained with HE, MT, and ORO. Samples were analyzed under polarized light to observe cholesterol crystal accumulation. (Scale bar = 50 μm). Black arrowhead: lipid droplet, white arrowhead: immune cell, green arrowhead: fibrotic region, yellow arrowhead: cholesterol crystal. Data are from one representative experiment of at least two independent experiments.

To investigate initiation of the steatotic and fibrotic liver phenotype, ALT and AST levels were measured daily in HCD-fed sB6 albino and B6 black. Representative mice from each group were sacrificed before HCD feeding (day 0) and at days 1 and 3, then analyzed via histological analysis. No morphological difference was observed between pre-HCD-induced B6 black and sB6 albino livers (Fig. 2a). After HCD feeding for 1-day, inflammatory cell infiltration and accumulation were observed in sB6 albino livers together with microvascular lipid and cholesterol crystal accumulation (Fig. 2b, yellow arrowhead). MT staining revealed no signs of fibrosis in either group (Fig. 2b). Large lipid droplet accumulation and inflammatory cells were observed after day 3 in sB6 albino (Fig. 2c, black arrowhead). Liver lipid accumulation was significantly higher than that in B6 black mouse livers, which only presented microvesicular lipid accumulation (Fig. 2c). Severe B6 albino livers contained high quantities of cholesterol crystals whereas B6 black showed no crystal accumulation (Fig. 2c, yellow arrowhead). Notably, HCD feeding for only 3-days initialized sB6 albino mouse liver fibrosis (Fig. 2c, green arrowhead). These observations correlate with the drastic increase in serum levels of liver injury markers ALT and AST from day 1 post-HCD feeding (Fig. 1b).

### HCD-fed sB6 albino mice exhibit altered serum lipoprotein profiles

**Figure 3:**
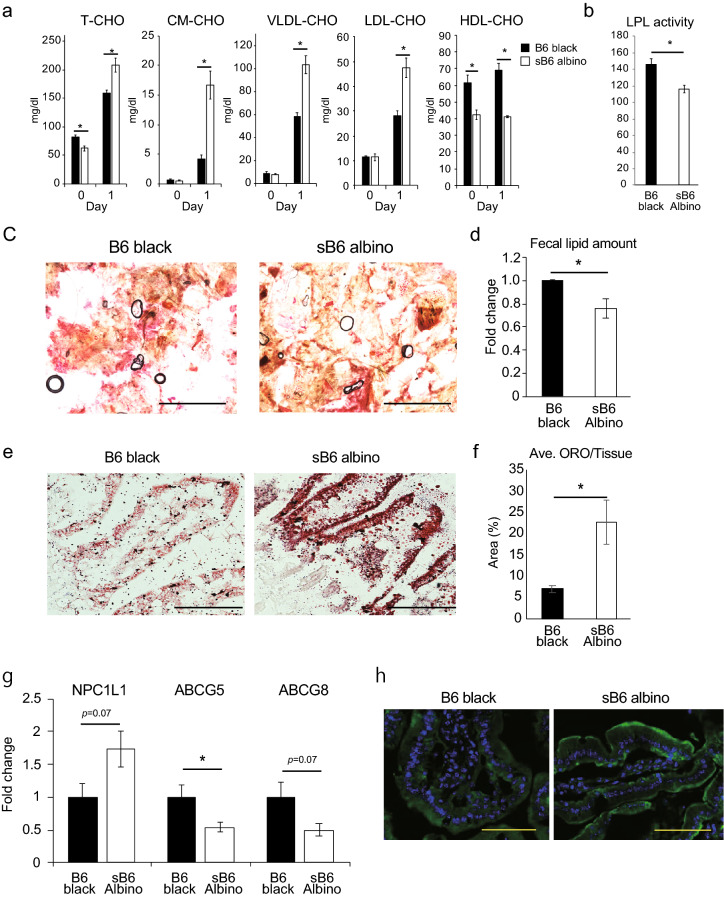
Severe B6 albino mice show impaired small intestinal cholesterol homeostasis. (**a**) Serum lipoprotein species before and 1-day after HCD feeding. T-CHO, CM-CHO, VLDL-CHO, LDL-CHO, and HDL-CHO are shown (B6 black n = 5, sB6 albino n = 5). (**b**) Serum LPL enzyme activity in sB6 albino (n = 3) and B6 black (n = 3) mice 1-day after cholesterol feeding. (**c**) Representative ORO staining of fecal matter from B6 black and B6 albino 1-day after HCD feeding (Scale bar = 1 mm). (**d**) Quantification of the lipid amount in feces normalized to B6 black amounts. Data from four independent experiments were pooled (B6 black n = 15, sB6 albino n = 18). (**e**) Representative view of ORO staining of SI 1.5 h after oral gavage with 2% cholesterol in 200 μl corn oil (Scale bar = 25 μm). (**f**) Quantification of ORO-positive area in intestinal tissue (B6 black n = 3, sB6 albino n = 3). (**g**) Relative expression of intestinal cholesterol transporter genes in HCD-fed B6 black and sB6 albino mice (B6 black n = 8, sB6 albino n = 11). Data were normalized using *36b4* mRNA levels. (**h**) Representative SI NPC1L1 staining (Scale bar = 100 μm). Data are from one representative experiment of at least two independent experiments and are presented as the means ± s.e.m. *p < 0.05 compared with B6 black (Welch’s t-test).

To understand how the underlying metabolic alterations lead to rapid initiation of liver inflammation and steatosis in HCD-fed sB6 albino, serum lipoprotein levels were measured in 10–12-week-old mice before and 1-day after HCD feeding. As in previous observations (Supplementary Fig. 1a), sB6 albino mice presented significantly lower serum T-CHO levels before HCD feeding, though significantly higher levels 1-day post-HCD feeding (Fig. 3a). No difference in serum chylomicron (CM-CHO) levels was observed before HCD induction. Both groups showed elevated levels 1-day after HCD feeding, but those in sB6 albino were significantly (four-fold) greater (Fig. 3a). Serum very low-density lipoprotein-cholesterol (VLDL-CHO) levels also did not differ before HCD but increased 5- and ten-fold in B6 black and sB6 albino, respectively, which differed significantly (Fig. 3a). At day 1, HCD feeding also increased serum low-density cholesterol (LDL-CHO) levels in both mouse groups but to a significantly higher degree in sB6 albino (Fig. 3a). Alternatively, serum high-density lipoprotein-cholesterol (HDL-CHO) levels did not significantly change following HCD feeding although sB6 albino showed significantly lower levels than B6 black at both time points (Fig. 3a). To understand the marked increase of serum CM-CHO and VLDL-CHO levels in HCD-fed sB6 albino, we focused on the lipid metabolism pathways of both lipoproteins. CM-CHO are absorbed to the blood in the intestines from digested exogenous cholesterol sources to become CM-CHO remnants, which enter the liver for further metabolism. LPL is the primary enzyme responsible for CM-CHO to CM remnant conversion and VLDL-CHO to IDL-CHO catalysis^25^. We therefore hypothesized that increased CM-CHO and VLDL-CHO levels could result from defective LPL enzyme activity in sB6 albino. To confirm this, we measured serum LPL activity in both mouse groups after 1-day of cholesterol feeding. Severe B6 albino showed significantly reduced serum LPL activity compared to B6 black (Fig. 3b), supporting our model.

### Severe B6 albino show impaired small intestinal cholesterol homeostasis

We next hypothesized that cholesterol homeostasis is altered in the sB6 albino mouse small intestines (SI). The SI plays an important role in cholesterol homeostasis by regulating exogenous cholesterol absorption and excretion^26^. We checked the status of SI cholesterol excretion in sB6 albino and B6 black by analyzing fecal lipid amounts. Mice were fed with HCD for 1-day and fecal matter was collected separately from each mouse. Fecal ORO staining revealed high lipid content in HCD-fed B6 black compared to sB6 albino mouse feces (Fig. 3c). Consistent with the ORO data, methanol chloroform fecal lipid extraction to quantify the lipid amount in feces demonstrated 24% lower fecal lipid content in sB6 albino (Fig. 3d), suggestive of impaired intestinal cholesterol excretion in these mice. To observe differences in dietary lipid absorption at the intestinal level, 10–12-week-old sB6 albino and B6 black were gavaged with oral cholesterol solution and SI samples were collected after 1.5 h and subjected to ORO staining. sB6 albino SI showed high ORO staining intensity, with quantification of ORO positive area revealing threefold larger positive area in sB6 albino than B6 black mouse SI (Fig. 3e,f), suggesting enhanced SI lipid absorption in HCD-fed sB6 albino. Cholesterol transporters present in the enterocyte luminal epithelium mainly regulate SI cholesterol homeostasis. We therefore evaluated the gene expression of intestinal cholesterol transporters NPC1L1 and ABCG5/ABCG8 in the HCD-fed mice groups. *Npc1l1* gene expression was nearly 1.5-fold higher in the sB6 albino group, whereas *Abcg5* and *Abcg8* gene expression was reduced by 50% (Fig. 3g), suggesting increased intestinal cholesterol absorption and defective reverse cholesterol transport in these mice. Consistent with this, anti-NPC1L1 staining in the SI revealed high NPC1L1 protein expression in the apical surface of enterocytes in sB6 albino compared to B6 black (Fig. 3h).

### The observed HCD-associated phenotypes are specifically caused by the G291T *Tyr* mutation in sB6 albino mice

**Figure 4:**
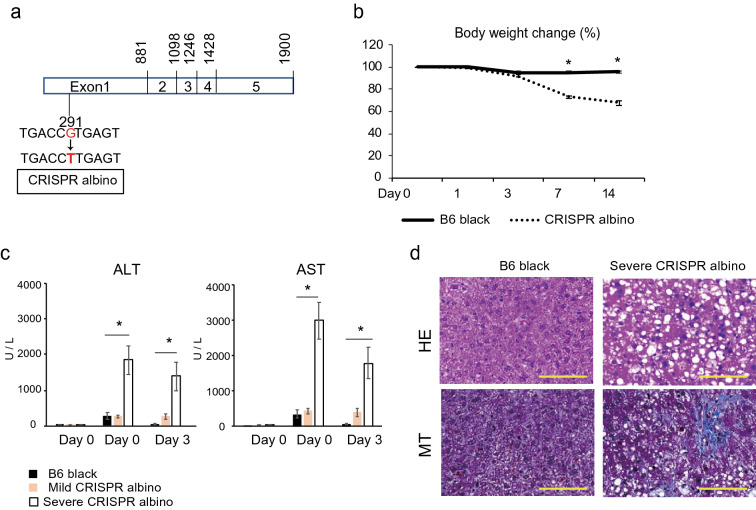
Mice with Tyrosinase c.291G > T mutation show the same phenotype to B6 albino with HCD feeding. (**a**) Tyrosinase transcript. CRISPR albino with C.291G > T mutation in Tyrosinase of C57BL/6J mouse using CRISPR/Cas9 system. (**b**) Transition graph of weight with HCD feeding in B6 black group and CRISPR albino group. (**c**) Serum ALT and AST values of B6 black group (black bar), mild CRISPR albino group (beige bar) and severe CRISPR albino group (white bar) with HCD feeding. (**d**) Liver histology of each group on day 14 of HCD feeding. Steatosis, inflammatory cell accumulation and fibrosis only observed in severe CRISPR albino livers. All the data are presented as the mean ± s.e.m. *p < 0.05 severe CRISPR albino (n = 7) compared with B6 black (n = 5) (Welch’s t-test).

To exclude the possibility that the observed phenotype results from some non-specific mutations in sB6 albino, we also employed a mouse carrying only the G291T mutation (Fig. 4a), generated using the CRISPR /Cas9 system^23^. Similar to B6 albino, 50% of the CRISPR-generated mice exhibited the same liver phenotype upon HCD feeding. HCD feeding for 2 weeks resulted in reduced body weight (Fig. 4b), elevated serum ALT and AST (Fig. 4c), and steatotic and fibrotic livers (Fig. 4d) in CRISPR albino mice. In comparison, Balb/c albino mice, which also display occlocutaneous albinism owing to the G369C single point mutation in the tyrosinase locus (Supplementary Fig. 5a) do not show the elevated liver inflammatory phenotype (Supplementary Fig. 5b–d).

## Discussion

In this study, we report that mutations in the tyrosinase gene may serve as a novel possible factor contributing to enhanced NAFLD/NASH susceptibility and underlie a previously undescribed rapid hepatic inflammatory phenotype in B6 albino. HCD feeding resulted in rapid onset of severe liver inflammation in sB6 albino compared to that in B6 black. Notably, inflammatory cell accumulation in the liver tissue and microvesicular steatosis in hepatocytes were observed after just 1-day of HCD feeding in sB6 albino. Moreover, 3-days of HCD feeding was sufficient to achieve a liver fibrotic phenotype in these mice. We therefore focused on further investigating the enhanced NASH susceptibility observed in B6 albino upon HCD induction.

A missense substitution, G291T (Arg77Leu), at the alternative 52 splice donor site for exon 1 of the *Tyr* gene has been shown to cause oculocutaneous albinism in pigmented C57BL/6J mice, resulting in the albino phenotype^27^. Although this constitutes the only recognized genetic difference between B6 black and B6 albino, the possibility exists that the observed phenotype may result from some non-specific mutations in B6 albino. However, we found that CRISPR/Cas9-generated mice carrying only the G291T mutation^23^ exhibited similar liver inflammatory phenotypes upon HCD feeding whereas Balb/c albino mice, which carry a G369C single point mutation in the tyrosinase locus^28^, do not. These results suggested that the G291T SNP in particular may lead to the observed NASH phenotype in B6 albino mice. Full-length Tyr mRNA has five exons and is matured by alternative splicing. Normally, the DNA sequence where splicing occurs is GT on the 5' side and AG on the 3' side, and the space between the two is spliced as an intron. In B6 albino, the splicing donor is lost due to G>T substitution. Moreover, in Balb/c, the splicing donor is lost because GT is changed to CT by G>C substitution^27^. Thus, the amino acids produced by the 78 bases from 291 to 369 nt in B6 albino may be related to the development of NASH. Kim et al. reported that the amino acid formed by this sequence is an epidermal growth factor (EGF)-like domain that contains a large amount of cysteine, which forms disulfide bonds^29^. Therefore, it is likely that B6 albino expresses an abnormal tyrosinase activity with an EGF-like domain.

Our results revealed elevated dietary cholesterol absorbance in HCD-fed sB6 albino mice, which was further supported by lower fecal lipid amounts. These observations suggested that the circulating cholesterol balance was disrupted by HCD load, with elevated amounts of cholesterol accumulating in the liver. Filipin staining and polarized light observation of liver tissue confirmed significantly high unesterified and crystalized cholesterol accumulation in sB6 albino livers. As cholesterol accumulation and crystallization in the liver can activate macrophage NLRP3 inflammasomes and trigger hepatic inflammation^30^, we concluded that the NASH induced in sB6 albino mice was triggered by inflammation consequent to hepatic cholesterol crystal accumulation.

Evaluation of serum lipoproteins in sB6 albino mice revealed significantly elevated serum CM-CHO levels upon 1-day HCD feeding resulting from altered SI cholesterol absorption. This suggested that sB6 albino mice exhibit poor CM-CHO uptake in the liver and/or decreased LPL activity or amount. However, the level of serum VLDL-CHO synthesized in the liver using the acquired CM-CHO was also increased on day 1, indicating normal liver uptake. Conversely, sB6 albino showed significantly lower LPL levels upon 1-day HCD loading and even before HCD feeding. These results may explain the elevated levels of serum CM-CHO and VLDL-CHO levels as low LPL results in less CM-CHO and VLDL-CHO hydrolysis. Furthermore, it has been reported that LPL activity is positively correlated with serum HDL-CHO levels^25^, explaining the low HDL-CHO content of the sB6 albino group even before HCD induction.

The tyrosinase gene is primarily expressed in retinal pigment epithelial cells of the eye, choroidal melanocytes, and hair follicle melanocytes of the skin in mammals^31^, although *Tyr* expression in liver has also been suggested^32^. In the present study, we demonstrated that *Tyr* is expressed at high levels in the SI in sB6 albino. This expression may influence the high NASH sensitivity in these mice by directly acting on the transporter related to cholesterol absorption or on bile acids involved in cholesterol digestion and absorption.

Normal tyrosinase is transported to the melanosomes by vesicles and associated with adapter proteins (AP) 1 and 3 present on the melanosome cell membrane^33^. Even though binding is weak, tyrosinase can bind to AP2 as well^34^. NPC1L1 also binds cholesterol on the microvillous membrane surface and is taken up into cells by endocytosis via AP2^35^. Based on these reports, we suspect that abnormal forms of Tyr may somehow maintain the anchoring activity of AP2 in endosomes, resulting in excessive cholesterol uptake by NPC1L1. The potential differences between responders (sB6) and non-responders (mB6) may be patterns of the expression of the alternative transcripts of Tyr mRNA, probably leading to an over-absorption of cholesterol. Nathalie et al. reported the effect of the c2j mutation in splicing variants. They described that the c2j mutation inactivated the splice donor site at nt 291 and enhanced the site's usage at nt 369. This resulted in a shift in the pattern of the expression of alternative transcripts^27^. There are four variants highly expressed in Tyr with the c2j mutation. Between them, two have Exon3/4, while the other two do not. If the frequency of the use of splicing donor sites depends on the individual, whether tyrosinase with Exon3 and 4 is dominant or not is also dependent on the individual. This would explain the appearance of about half sB6 and half mB6 in our model. The experimental proof of this idea will require in-vitro experiments using intestinal epithelial cells with abnormal Tyr produced by the c2j mutation. Whether or not AP2 binds to Tyr and whether or not this increases the activity of NPC1L1 will be the subject of future studies. Tyrosinase is essentially an enzyme that oxidizes monophenols, so that it is thought to have various substrates. We cannot exclude the possibility of other effects of tyrosinase in cholesterol metabolism. We believe that further analysis of these issues will be necessary in the future.

Several experimental animal models for NASH have been reported involving genetic, dietary, and toxic interventions. Notably, our HCD-fed B6 albino NASH mouse model exhibits competitive advantages compared to existing models in regarding speed of achieving the complete fibrotic phenotype. Genetically modified models such as leptin-deficient mice develop steatosis, but further feed alterations are required to obtain the fibrotic phenotype^36^. SREBP1c overexpressing or phosphatase tensin homolog (PTEN)-deleted mice exhibit steatosis with normal diet albeit only mild fibrosis^37^. Additionally, these models all require long induction times of around 20 weeks^38^. The methionine choline-deficient (MCD) dietary model is still the most commonly used for its simplicity and short induction duration of around 3–5 weeks. In comparison, our HCD-fed B6 albino model offers the advantage of starting inflammation and steatosis at day 1 of HCD feeding and fibrosis from day 3, proceeding to full NASH liver profile in just in 2 weeks. Thus, the HCD-fed B6 albino model may represent the most rapid NASH mouse model introduced to date.

A study in the US has shown differences in NAFLD/NASH prevalence among people of different races and ethnic groups. After excluding subjects suspected of having hepatitis C and a history of excessive alcohol consumption, NAFLD prevalence was 24.1% in Hispanics, 17.8% in non-Hispanic whites, and 13.5% in non-Hispanic blacks^18^. In a separate cohort study of 12,454 individuals by Lazo et al., Hispanics had the greatest prevalence among Americans, and non-Hispanic blacks had the lowest^39^. This racial difference in susceptibility to NASH is thought to be due to genetic factors as well as economic and environmental reasons. Iwata et al. found that tyrosinase activity in black skin samples was three times higher than that in white skin samples^40^. Tyrosinase activity in human skin correlated with the melanin content in melanocytes^40^.

The B6 albino used in this study has a mutation in the tyrosinase gene. As B6 albinos have a white color, the tyrosinase activity must diminish. However, there has not been any report that human albinos with a complete loss of tyrosinase activity develop NASH, and with another white strain, Balb/c mice do not develop NASH (Supplementary Fig. 5). Therefore, it is unlikely that a simple tyrosinase deficiency causes NASH. Future analysis of mice with human tyrosinase mutations will be necessary.

## Methods

### Mice and experimental diet

B6 (CG)-*Tyr*^*c-2j*^/J (B6 albino) (Jackson Laboratory, Bar Harbor, Maine, USA), G291T mutation in *Tyr* gene mice generated using the CRISPR /Cas9 system^23^, C57BL/6J (B6 black) mice and Balb/c mice (8–12 weeks old) were bred in a specific pathogen free area in the Animal Resource Center of the University of Tsukuba, maintained at 23.5 ± 2.5 °C and 52.5 ± 12.5% humidity with 14/10 h light/dark period. Food and water were supplied ad libitum. HCD (1.25% cholesterol; Oriental Yeast Co. Ltd., Japan) was used.

### Ethical approval

All experiments were performed in compliance with relevant Japanese and institutional laws and guidelines and approved by the University of Tsukuba animal ethics committee (approval number 18-228). And the study was carried out in compliance with the ARRIVE guidelines (https://arriveguidelines.org/arrive-guidelines).

### Serum analysis

Blood was collected from the facial vein under isoflurane anesthesia, held at 25 °C for 30 min, and centrifuged (800×*g*, 4 °C for 30 min) for serum separation. Serum alanine aminotransferase (ALT) (#3250), aspartate aminotransferase (AST) (#3150), total cholesterol (T-CHO) (#1450), and glucose level (#1050) were measured using a Fuji Dry Chem 7000 V (all from FUJIFILM, Japan).

### Histological analysis

Dissected organs were fixed in 4% paraformaldehyde for 4–5 h and incubated in 30% sucrose at 4 °C overnight. Fixed organs were frozen in optimal cutting temperature embedding medium (Sakura Finetek, Japan) and 5-μm sections prepared at − 20 °C using a cryotome (Leica, Germany). Organs for paraffin blocks were fixed in Mildform 10N (Wako, Japan) overnight, embedded in paraffin, and sectioned using a MicromHM-335 E microtome (Microm International GmbH, Walldorf, Germany). Hematoxylin and eosin (HE), Masson trichrome (MT), and Oil Red O (ORO) staining were performed per established protocols. For immunohistochemical analysis of Mac2 expression in liver macrophages, frozen sections were incubated with a 1:200 dilution of rat anti-mouse anti-Mac2 antibody (Cedarlane, Canada), then Alexa Fluor 488-conjugated chicken anti-rat IgG secondary antibody (1:500 dilution; Thermo Fisher Scientific, MA) for secondary fluorescence staining. Finally, sections were mounted with Fluoromount (CosmoBio, CA) containing 2 μg/mL Hoechst and observed using a BIOREVO BZ-9000 microscope (Keyence, Japan).

### Observation of cholesterol crystals

A polarizing plate was placed between the light source of the BIOREVO BZ-9000 microscope and the specimen slide. The polarizing plate was rotated and positioned to acquire the polarized light beam. Observation was performed using unstained frozen specimens.

### Quantitative reverse transcription-polymerase chain reaction (RT-qPCR)

Total RNA was extracted from the frozen livers using the Qiagen RNeasy Kit (QIAGEN, The Netherlands) and cDNA prepared via reverse transcription using the QuantiTect Reverse Transcription Kit (QIAGEN). PCR was performed using a SYBR green PCR master mix (TOYOBO, Japan). Primer sequences are shown in Table 1. The relative expression levels of *Il1b*, *Tgfb*, and *Col1a1* were normalized using *Hprt* and those of *Npl1c1*, *Abcg5*, and *Abcg8* using *36b4*.

**Table 1:** Primers used in this study.

Gene	Primer sequence
*Hprt*	F: 5′-CAAACTTTGCTTTCCCTGGT-3′
	R: 5′-CAAGGGCATATCCAACAACA-3′
*Il1b*	F: 5′-CAACCAACAAGTGATATTCTCCATG-3′
	R: 5′-GATCCACACTCTCCAGCTGCA-3′
*Tgfb*	F: 5′-CCTGAGTGGCTGTCTTTTGACG-3′
	R: 5′-AGTGAGCGCTGAATCGAAAGC-3′
*Col1a1*	F: 5′-CCTCAGGGTATTGCTGGACAAC-3′
	R: 5′-ACCACTTGATCCAGAAGGACCTT-3′
*36b4*	F: 5′-GAGACTGAGTACACCTTCCCAC-3′
	R: 5′- ATGCAGATGGATCAGCCAGG-3′
*Npc1l1*	F: 5′-TTGCCTTGACCTCTGGCTTAG-3′
	R: 5′-AGGGCGGATGAATCTGTGC-3′
*Abcg5*	F: 5′-TCAGGACCCCAAGGTCATGAT-3′
	R: 5′-AGGCTGGTGGATGGTGACAAT-3′
*Abcg8*	F: 5′-GACAGCTTCACAGCCCACAA-3′
	R: 5′-GCCTGAAGATGTCAGAGCGA-3′

### Serum lipoprotein analysis

Serum obtained as described above was sent to Skylight Biotech Co., Ltd. for lipoprotein analysis without breaking the cold chain.

### Lipoprotein lipase (LPL) activity assay

LPL activity was measured from serum obtained by centrifugation using a Lipoprotein Lipase Activity Assay Kit (CellBiolabs, CA). Serum LPL incubation with a fluorescent substrate allows probe cleavage, emitting fluorescence. Fluorescence (485 nm wavelength) was measured using a microplate reader (Thermo Fisher Scientific).

### Fecal lipid quantification

At 24 h after HCD feeding, all feces in the cages were collected and analyzed as previously described^41^. Briefly, powdered feces (1 g/mouse) was collected into 15 mL falcon tubes, then 5 mL saline and 5 mL chloroform/methanol mixture (2:1) added, mixing well at each step. The mixture was centrifuged (400×*g*, 10 min, 25 °C) for separation into three layers; the bottom layer contained the extracted lipid layer, which was collected using a 22G needle and 5 mL syringe into a glass tube. Chloroform/methanol was evaporated in a draft chamber for 3-days to obtain extracted lipid.

### SI cholesterol absorption analysis

Cholesterol absorption analysis was performed as previously published^42^. Briefly, mice were fasted overnight, fed with 200 μL of 2% (p/v) cholesterol (Sigma-Aldrich) in corn oil or 200 μL H_2_O for controls, and sacrificed at 90 min after gavage. The duodenum and proximal jejunum were collected, washed in phosphate-buffered saline to remove external lipids, and transversal frozen tissue slices (5 mm) obtained by cryo-sectioning at − 20 °C. The neutral lipids of intestinal were stained using Oil red O as previously described^43^.

### Statistical analysis

All quantified data are presented as the means ± s.e.m. The data reflect one representative experiment of at least two independent experiments. Probability values were calculated using Welch’s t-test. Results were considered statistically significant at *p* values < 0.05.

## Supplementary Information

Supplementary Information.
