# Supplementary material for: Albino mice with the point mutation at the tyrosinase locus show high cholesterol diet-induced NASH susceptibility
Source: Sci Rep. 2021 Nov 8;11:21827. doi: 10.1038/s41598-021-00501-5 (PMC8576022; doi:10.1038/s41598-021-00501-5)
Supplement: Supplementary file 1 — Supplementary Information. [file 41598_2021_501_MOESM1_ESM.pdf]

Supplemental figure 1

a

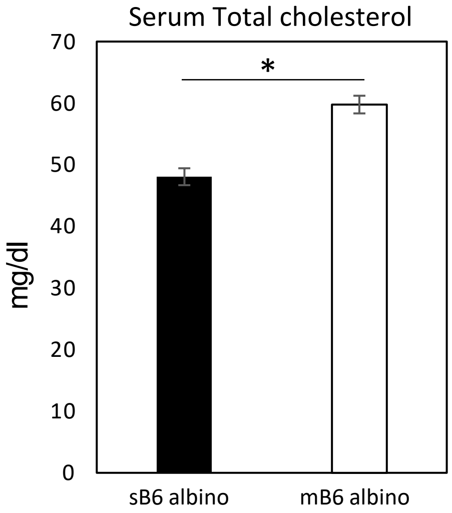

b

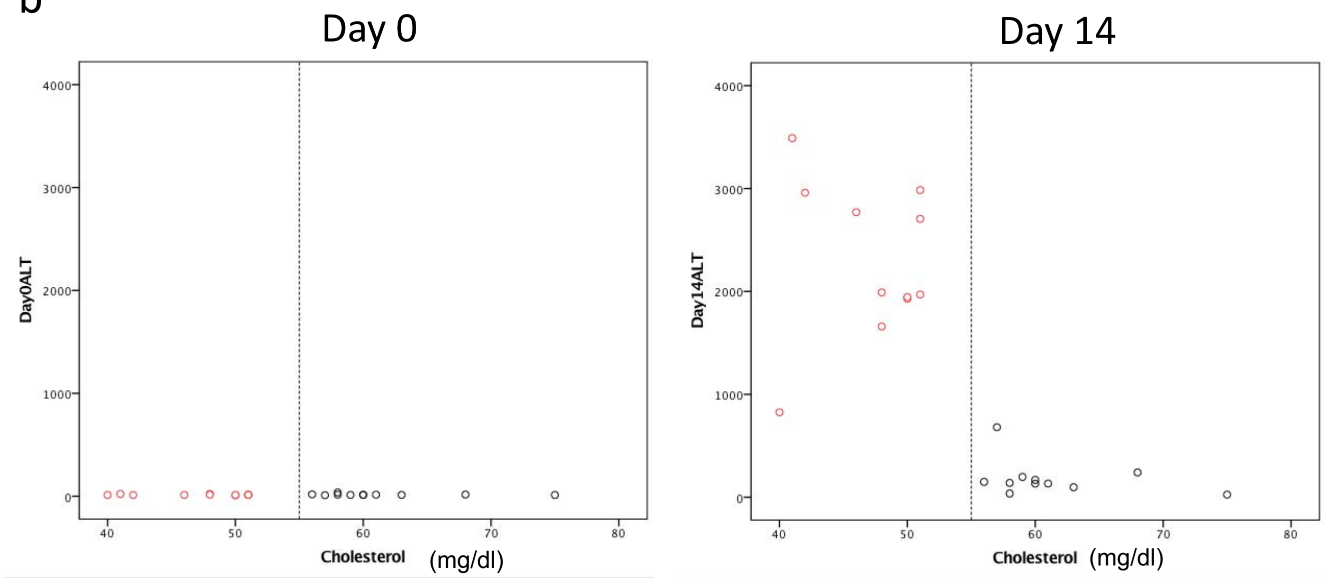

Correlation is significant at the 0.01 level (2-tailed)

Supplemental Figure 1: Severe phenotype of HCD fed albino mice is related to pre-induction low serum cholesterol levels

a) Serum total cholesterol levels before HCD induction in two B6 albino mice groups, which develop into severe (n = 11) and mild (n = 11) phenotype with HCD feeding.

b) Correlation between serum total cholesterol levels and serum ALT levels in each B6 albino mice (n=11) before HCD feeding in day 0 (left panel) and after 14 days of HCD feeding (right panel). It is clear that day 0 low serum total cholesterol mice show significantly high levels of Serum ALT indicating severe liver damage. All the data are presented as the mean ± s.e.m. *$p < 0.05$ sB6 albino compared with mB6 albino (Welch's t-test).

Supplemental figure 2

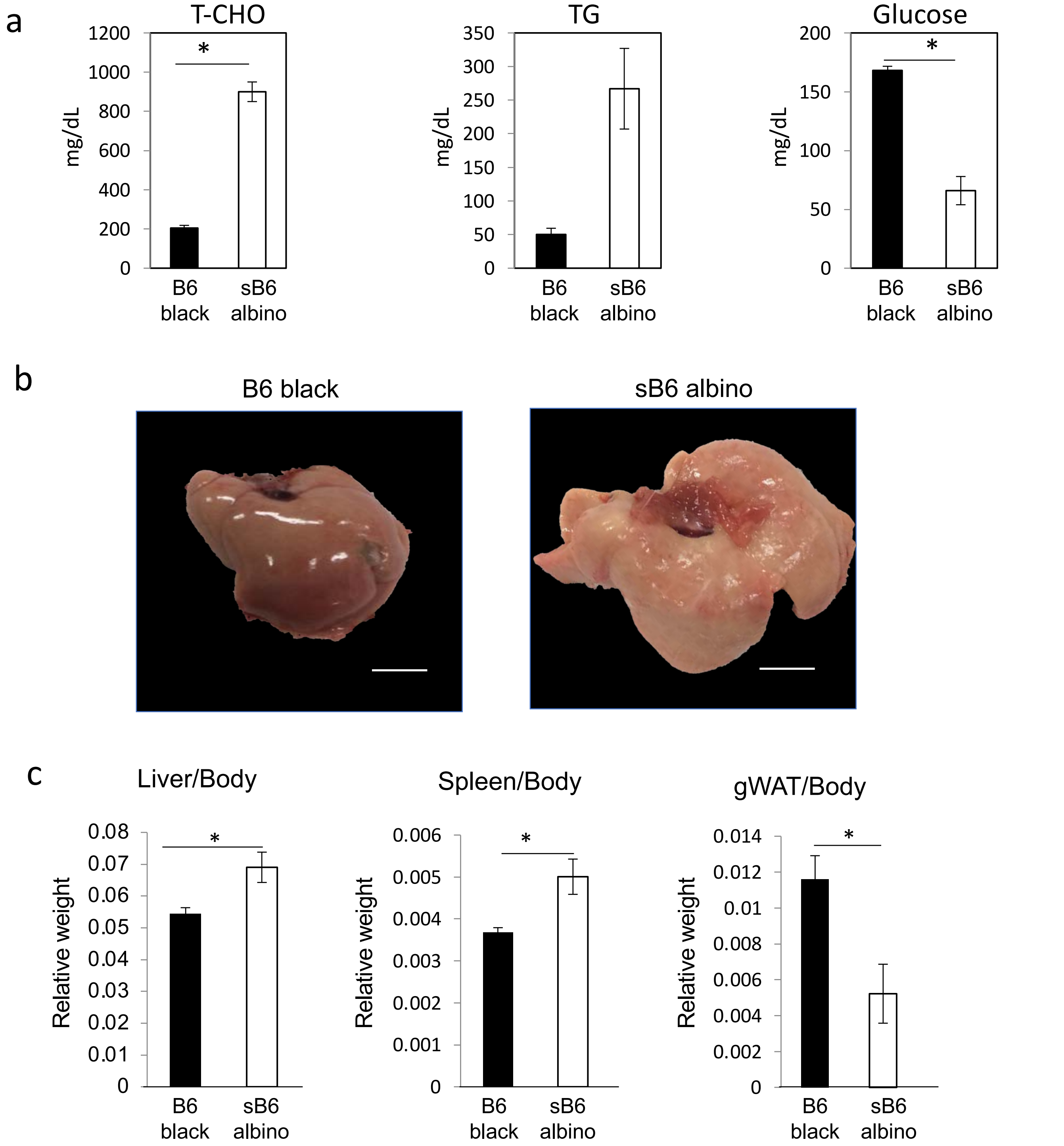

Supplemental figure 2: Ten weeks HCD fed albino mice showed serum metabolic parameter changes and signs of liver injury

a) Serum total cholesterol, triglyceride and glucose levels of 10 weeks HCD fed B6 black (n=9) and sB6 albino mice (n=3). b) Macroscopic view of liver from B6 black and sB6 albino mice after 10 weeks of HCD feeding.
c) Relative organ weights of liver, spleen, and gonadal white adipose tissue after 10 weeks of HCD fed B6 black (n=9) and sB6 albino mice (n=3). The data are from one experiment that was representative of at least two independent experiments. The data are presented as the mean ± s.e.m. *$p < 0.05$ compared with B6 black group (Welch's t-test).

Supplemental figure 3

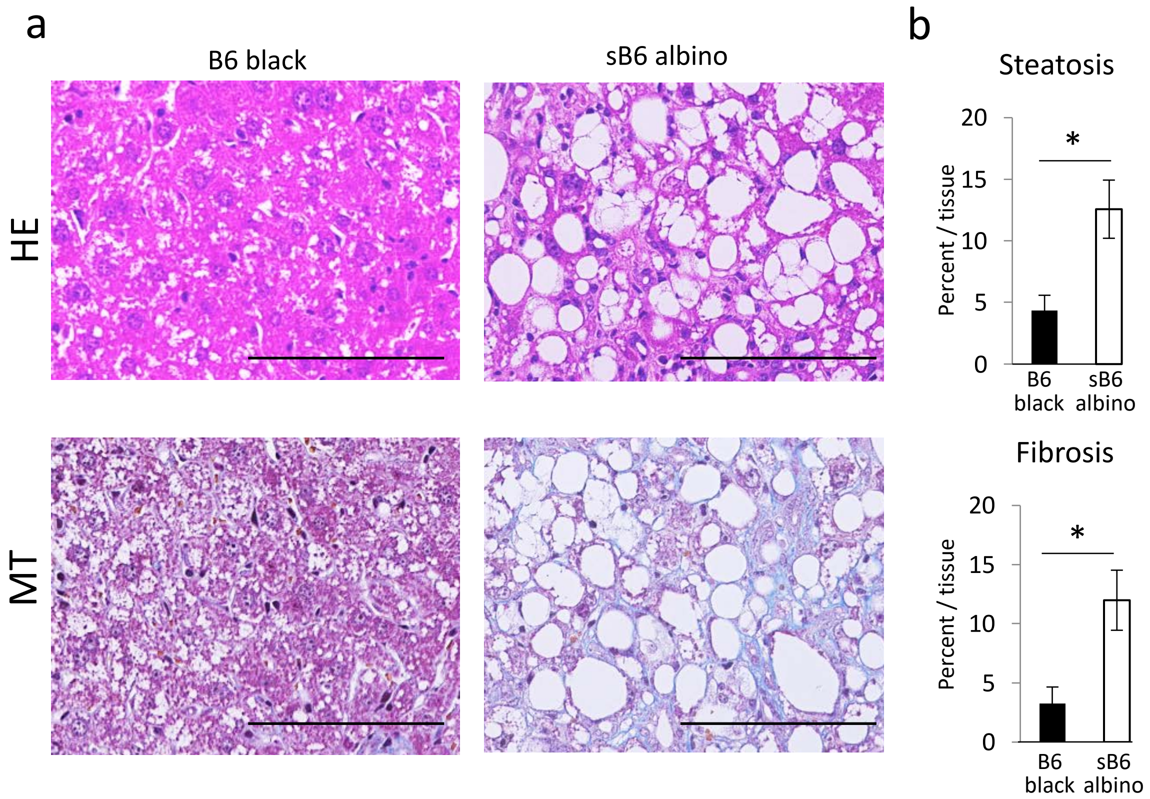

Supplemental figure 4: Ten weeks of HCD feeding induced liver steatosis and fibrosis in B6 albino mice

a) Top panel, Hematoxylin and Eosin staining and bottom panel, Masson Trichrome staining of livers of 10 weeks HCD fed B6 black and sB6 albino mice. Scale bar: 100μm

b) Quantification of relative area of steatosis and fibrosis in B6 black (n=9) and sB6 albino (n=3). The data are from one experiment that was representative of at least two independent experiments. The data are presented as the mean ± s.e.m. *$p < 0.05$ compared with WT, (Welch's t-test).

Supplemental figure 4

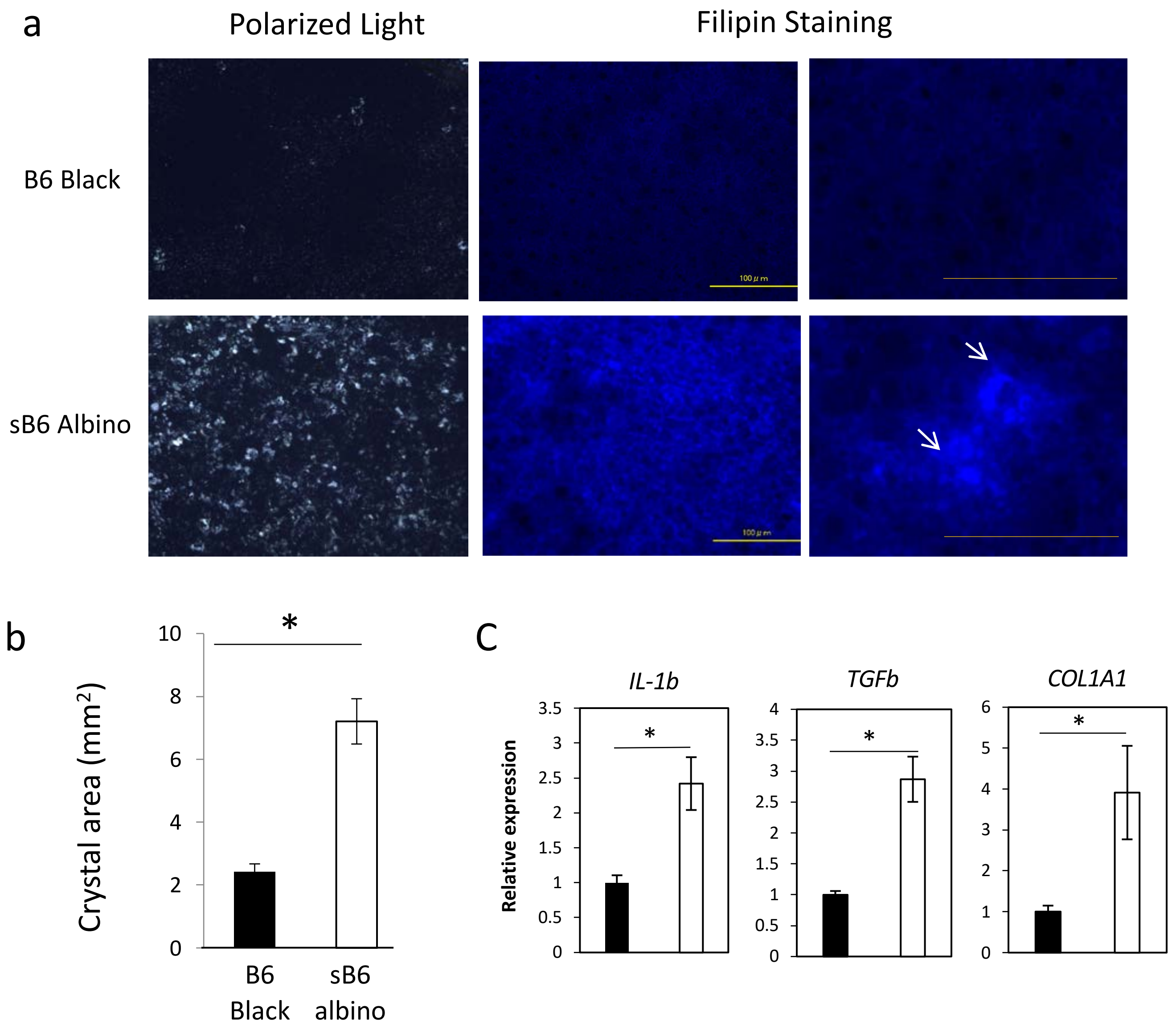

Supplemental Figure 4 : B6 albino mice liver tissues show significantly high cholesterol accumulation with 2 weeks of HCD feeding.

a) 2 weeks HCD fed sB6 albino and B6 black mice liver tissues, left panel; under polarized light (cholesterol crystals in white color) and middle and right panel with Filipin staining (unesterified cholesterol in blue).

b) Quantification of cholesterol crystal area in liver tissue in each group after 2 weeks of HCD feeding showed significantly high crystal accumulation in sB6 albino livers.

c) e Relative mRNA expression of IL-1β, TGFβ, and COL1A1 in the liver (B6 black n = 8, sB6 albino n = 11). Expression levels were normalized using Hprt mRNA. All the data are presented as the mean ± s.e.m.
*p < 0.05 sB6 albino compared with B6 black (Welch's t-test).

Supplemental figure 5

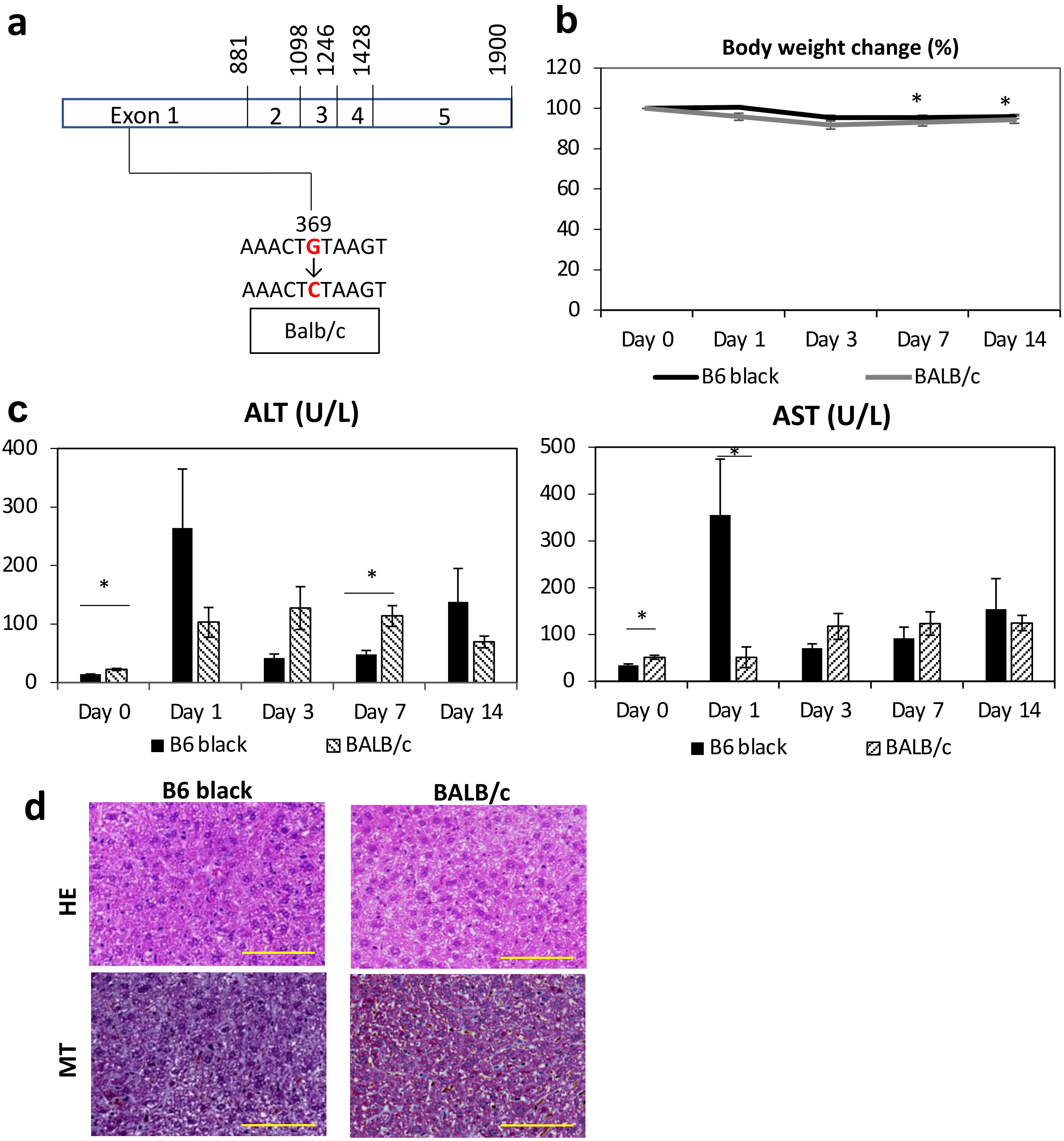

Supplementary Figure 5: Balb/c mice with Tyrosinase c.369 G> C mutation do not show any liver inflammatory phenotype with HCD feeding .

a) Balb/c Tyrosinase transcript with C.369 G>C mutation.

b) Transition graph of weight with HCD feeding in B6 black group and Balb/c group with HCD feeding.

c) Serum ALT and AST values of B6 black group (Black bar) and Balb/c group (dotted bar) with HCD feeding.

d) Liver histology of each group on day 14 of HCD feeding. No signs of liver damage was observed in any group. (B6 black: n = 5, Balb/c: n = 10) All the data are presented as the mean ± s.e.m. *$p < 0.05$ Balb/c compared with B6 black (Welch's t-test).
